# Highly cross-linked polyethylene versus conventional polyethylene in primary total knee arthroplasty: comparable clinical and radiological results at a 10-year follow-up

**DOI:** 10.1007/s00167-022-07226-6

**Published:** 2022-11-21

**Authors:** Fortunato Giustra, Alessandro Bistolfi, Francesco Bosco, Nicolò Fresia, Luigi Sabatini, Paola Berchialla, Veronica Sciannameo, Alessandro Massè

**Affiliations:** 1 Department of Orthopaedics and Traumatology, University of Turin, CTO, Via Zuretti 29, 10126 Turin, Italy; 2 Orthopaedics and Traumatology, Ospedale Cardinal Massaia Asti, Via Conte Verde 125, 14100 Asti, Italy; 3 Department of Clinical and Biological Sciences, University of Turin, Turin, Italy

**Keywords:** Polyethylene, CPE, HXLPE, Crosslinked, Knee, Total knee arthroplasty, Retrospective

## Abstract

**Purpose:**

Highly crosslinked polyethylene (HXLPE) was introduced in total knee arthroplasty (TKA) to reduce wear and consequent revisions for loosening due to conventional polyethylene (CPE) wear. This study aims to analyse whether HXLPE is as safe as CPE and could improve the TKA clinical and radiological results in a long-term follow-up.

**Methods:**

This retrospective study included all consecutive starting series of 223 patients with severe primary knee osteoarthritis (OA), with a minimum follow-up of 10 years treated between July 1st, 2007, and July 31st, 2010. After excluding patients who did not respect the inclusion and exclusion criteria, 128 patients were included in the analysis of this study. The patients were then divided into two groups according to the type of polyethylene (PE) implanted: CPE or HXLPE liners. All patients were evaluated for clinical and radiological parameters, causes and revision rates related to the type of PE implanted.

**Results:**

HXLPE appears to be as safe as CPE in TKA, reporting no higher revisions for osteolysis, prosthesis loosening, infection, and mechanical failure. Nevertheless, no statistically significant differences were found between the two groups in the clinical and radiological outcomes evaluated.

**Conclusions:**

Clinical, radiological results, and revision rates are similar between HXLPE and CPE in TKA after 10 years of follow-up, although HXLPE benefits remain controversial.

**Level of evidence:**

III.

## Introduction

New surgical techniques and materials have been developed to improve the longevity and performance of prosthetic implants in total knee arthroplasty (TKA) [2, 3, 24, 25]. Several studies demonstrated aseptic loosening caused by osteolysis as the main cause of long-term TKA failure [12, 29]. Polyethylene (PE) wear and debris may activate an inflammatory response with subsequent bone resorption leading to osteolysis [2, 3]. The incidence of osteolytic lesions after TKA is between 5% and 20%, with follow-up between 5 and 15 years. [15]. Highly crosslinked polyethylene (HXLPE) has been introduced in TKA to minimise debris generation and the subsequent inflammatory response responsible for osteolysis [1–3]. Despite encouraging results of HXLPE over conventional polyethylene (CPE) in TKA in laboratory studies [9, 22], the same efficacy has not been demonstrated in short- to the medium-term clinical application [10, 23], and issues remain about the mechanical properties in TKA [3, 4].

The objective of the study was to compare the clinical and radiological outcomes of patients after primary TKA using a CPE or HXLPE liner to evaluate whether the cross-linked innovation really provided long-term benefits in TKA in terms of wear-related osteolysis, safety, cost, and re-operations.

## Materials and methods

A retrospective study was carried out on a consecutive starting series of 223 patients with severe primary knee OA in whom a primary TKA was implanted at our Orthopaedics and Trauma Department between July 1st, 2007, and July 31st, 2010.

### Inclusion and exclusion criteria

Patients lost to follow-up, died before reaching a follow-up interval of at least 10 years, suffering from systemic or local infectious diseases, having a history of corrective osteotomy, ligament reconstruction, or previous severe traumatic surgical treatment around the knee were excluded. Therefore, after excluding patients who did not respect the inclusion and exclusion criteria, 128 patients were included in the analysis of this study. The included patients were then divided into two groups according to the type of PE implanted. Until December 2008, the CPE insert was implanted in all treated patients. Since January 2009, due to the encouraging results of both in vivo and in vitro studies, the HXLPE insert was used in all TKAs [3, 9, 22]. All patients underwent NexGen^®^ complete knee solution Legacy^®^ knee-posterior stabilised (LPS) Zimmer TKAs performed with CPE or HXLPE inserts. The design and materials of the tibial component (titanium alloy [Ti-6V-4Al]) and femoral component (cobalt–chromium–molybdenum [Co–Cr–Mo] alloy) were the same in both groups. The only difference was the type of PE used in the implant, either CPE or HXLPE.

### Surgical technique

An anteroposterior (AP), a lateral knee weight-bearing view, a Rosenberg view, and a Merchant–Lauren view were performed for preoperative radiographic planning [25]. The same experienced knee surgeons performed the surgical procedure and used a mechanical alignment technique for all TKAs included in the present study. The mechanical alignment resulted in a change in the preoperative knee phenotype in most cases. However, this would not appear to result in clinically significant differences between patients with and without preoperative coronal knee alignment, as described by Sappey-Marinier et al. in their study [26]. An anterior midline skin incision was made, followed by a medial parapatellar capsular incision. After removing the osteophytes and excision of the anterior and posterior cruciate ligaments, the distal cut was made on the femur using an intramedullary rod in the medullary canal. The AP cut of the distal part of the femur was performed using an anterior reference guide. AP and chamfers femoral resections were performed by positioning the 4-in-1 cutting guide on the femur. The correct position of the femoral component was determined using the trans epicondylar axis, the Whiteside line, and by giving three degrees of external rotation to the femoral component relative to the posterior aspect of the condyles. Subsequently, the cut on the tibia was made on its perpendicular axis. The ligaments were balanced to obtain an equal knee flexion and extension gap. The patella was reshaped if deformed by arthritis, if the erosion of the articular cartilage exceeded 50% of the surface area (Outerbridge grade III or IV) [30], if a malposition of the patella before surgery or after implantation of the femoral or tibial components was observed. All implants were cemented after irrigation with pulsed washing, drying, and pressurisation of the vacuum-mixed cement. Post-operatively, the patients started on active knee range of motion (ROM) exercises and were given weight-bearing, protected by two crutches for 40 days. Patients were evaluated with a minimum follow-up of 10 years.

### Data extraction

For each patient, demographic data were recorded and included in a standard template: sex, age at the time of surgery, and body mass index (BMI). At the final follow-up, all patients were assessed for clinical, and radiological parameters related to the type of polyethylene implanted. Clinical outcomes were evaluated according to ROM, Knee Society Score (KSS) knee score, and KSS function score, collected before and after surgery [21]. The KSS knee and function scores are a valid and internally consistent measure of TKA outcomes, because they are applicable across age, activity level, sex, and implant type, confirming internal reliability and analysed for differential item functioning [28]. Specific data on prosthetic implant failure were recorded and analysed: re-intervention, re-intervention for prosthesis loosening, re-intervention for infection, and mechanical failure. All the post-operative radiographs were evaluated for the presence of radiolucent lines and osteolysis. An implant was defined as loose when there was evidence of component migration and/or circumferential translucent lines thicker than two millimetres in each area [27]. The authors (FG, FB, and NF) collected all information using a standard proforma with direct patient examination, clinical evaluation tables, and telephone interviews. In case of discrepancies, an experienced knee surgeon (AB) was consulted to resolve any further uncertainties.

### Ethical approval

The institutional Review Board (IRB) of the author’s institution defined this study as exempt from IRB approval (retrospective study on a well-established surgical procedure) and was conducted in accordance with the ethical standards laid down in the 1964 Helsinki Declaration and its later amendments. All patients were informed about the study and consented to participate.

### Statistical analysis

All data were analysed using R software, version 4.0.5 (2020; R Core Team, Vienna, Austria). A descriptive statistical analysis was performed for the population cohort’s demographic, clinical, and radiological data. A two-sized Mann–Whitney *U* test, at the significance level (alpha) 0.05, adjusted for multiplicity with Bonferroni correction, was used to detect sample size calculation. Median values and interquartile range (IQR) were calculated for continuous variables. Absolute frequencies and percentages were calculated for categorical variables. Quantitative variables were analysed with the Mann–Whitney *U* test, while qualitative variables were analysed with the chi-square test and Fisher’s exact test. Multivariate linear regression was then performed to examine the KSS knee score post-operative and the KSS function score post-operative. Finally, a *p* value < 0.05 was considered statistically significant.

## Results

**Fig. 1:**
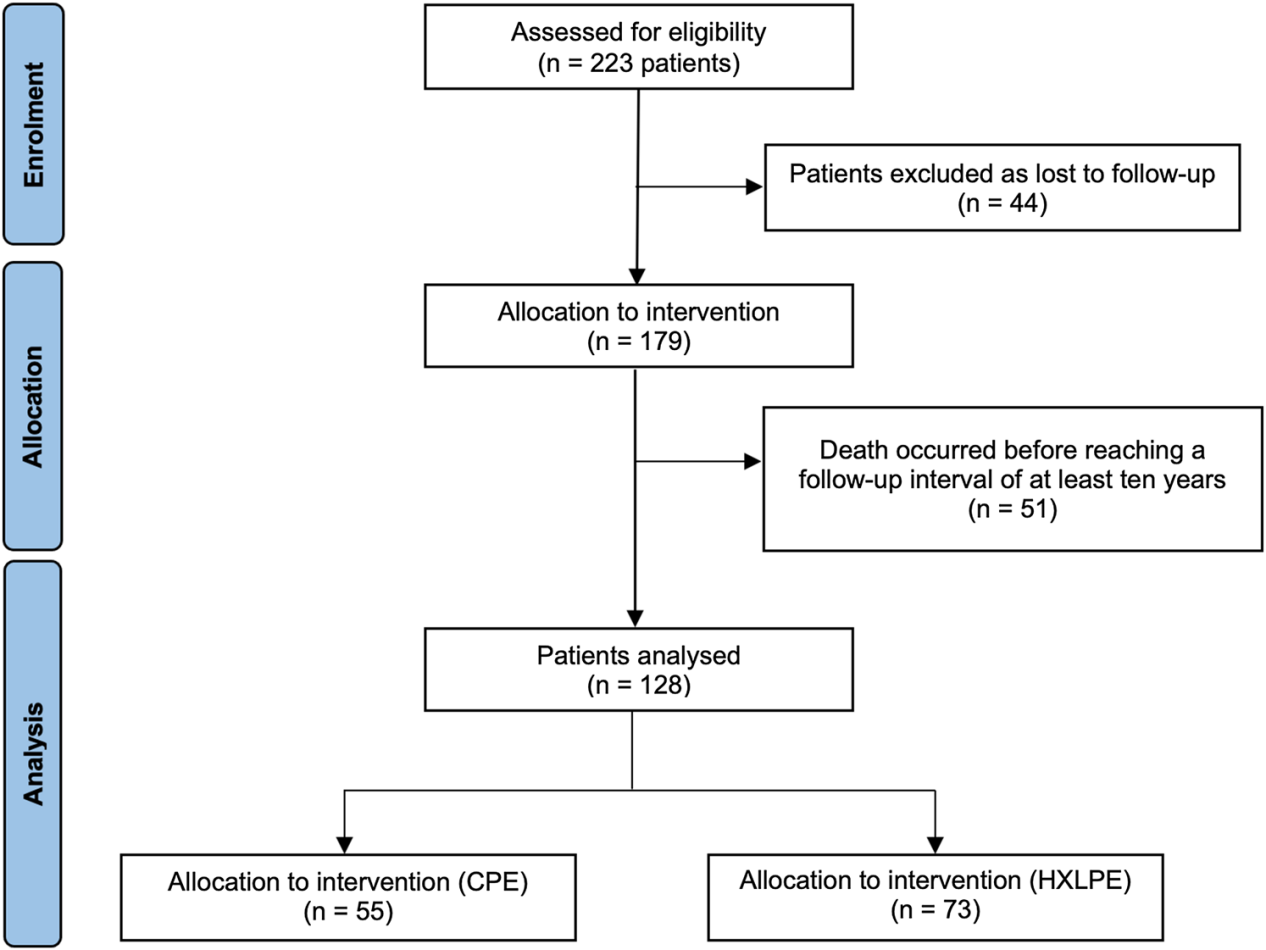
Consort flow diagram of study: all patients. *n* number of evaluation cases, *CPE* conventional polyethylene, *HXLPE* highly crosslinked polyethylene

A total of 128 patients, 55 treated with CPE and 73 with HXLPE liners, were analysed with a minimum follow-up of 10 years (Fig. 1). The median follow-up was 150 (range 135–171) months. The main demographic patients’ characteristics, such as the median age at the time of the surgical procedure, BMI value, and male/female percentages, were reported in Table 1.

**Table 1:** Main demographic characteristics of patients collected

	Total patients	CPE	HXLPE	*p* value
*N*°	128	55	73	*p*
Age at the time of surgery (median [IQR])	71.50 [66.00, 76.00]	73.00 [69.00, 76.00]	70.00 [64.00, 75.00]	< 0.05
BMI (median [IQR])	28.50 [26.20, 31.20]	27.70 [25.90, 30.70]	28.90 [26.55, 31.30]	> 0.05
Sex = M (%)	32 (25.00)	12 (21.82)	20 (27.40)	> 0.05

*CPE* conventional polyethylene, *HXLPE* highly crosslinked polyethylene, *N*° number of evaluation cases, *p p* value, *IQR* interquartile range, *BMI* body mass index, *M* male, *%* percentage

### Patient‐reported outcome measures (PROMs)

**Fig. 2:**
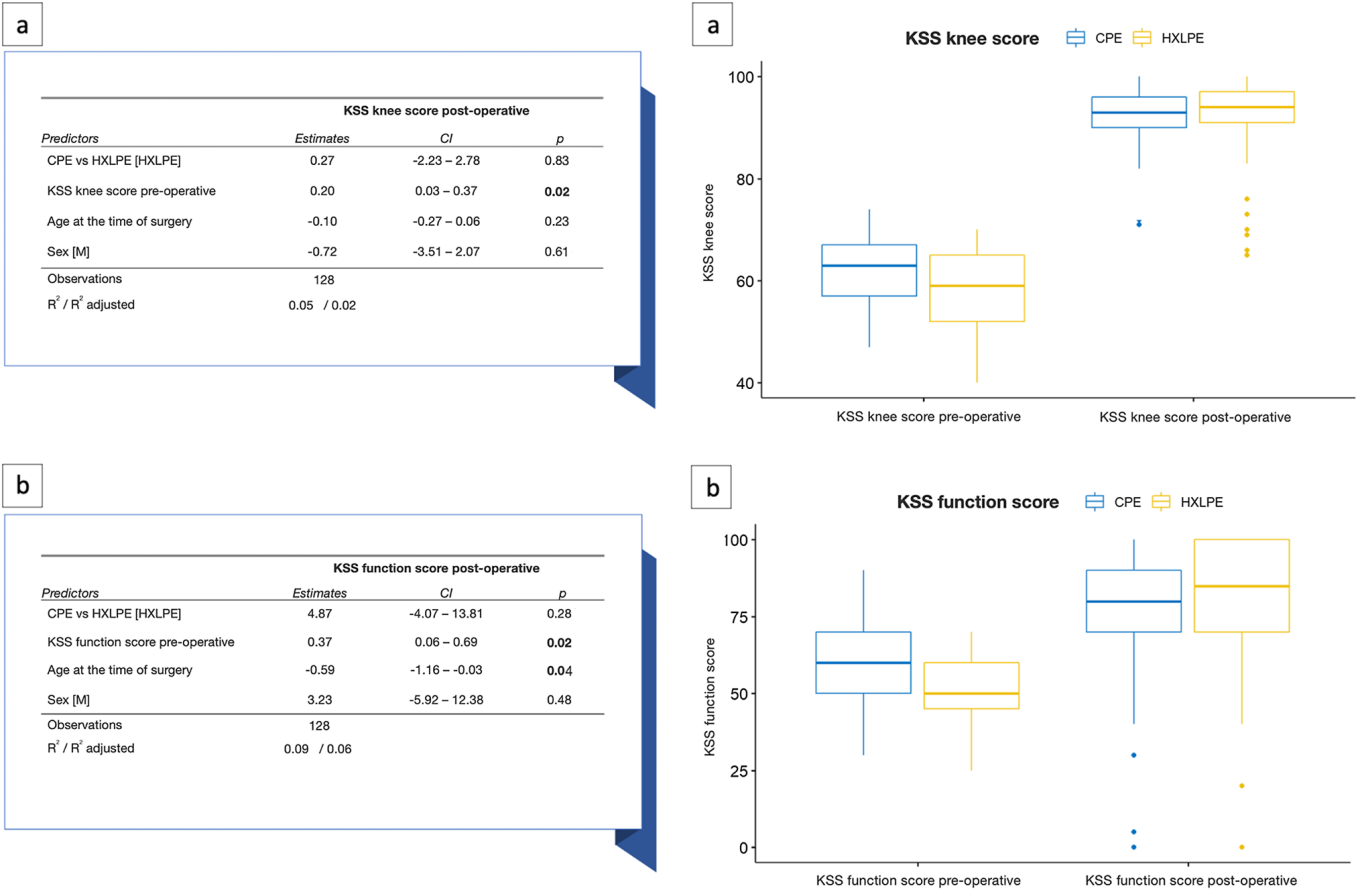
Multivariate linear regression was performed to examine the KSS knee score post-operative (**a**) and the KSS function score post-operative (**b**) in patients treated with HXLPE or CPE. *KSS* Knee Society Score, *CI* confidence interval, *p p* value, *CPE* conventional polyethylene, *HXLPE* highly crosslinked polyethylene, *M* male

No statistically significant differences were found between the two polyethylene groups in the post-operative ROM, KSS knee and KSS function scores (Table 2). Based on the multivariate linear regression analysis, the HXLPE group reported average post-operative KSS knee and function scores higher than CPE one, although not statistically significant (Fig. 2). The sample size calculation analysis showed that the sample size of the groups reaches 80% power to detect a difference of 8 ± 14 points in the KSS knee and function scores.

**Table 2:** Clinical, functional, and radiographic parameters in total knee arthroplasty (TKA) comparing highly crosslinked polyethylene (HXLPE) to conventional polyethylene (CPE)

	Total patients	CPE	HXLPE	*p* value
*N*°	128	55	73	
Patient‐reported outcome measures (PROMs):				
ROM pre-operative (median [IQR])	110 [100, 115]	110 [100, 110]	110 [100, 120]	
ROM post-operative (median [IQR])	110 [104, 110]	110 [100, 110]	110 [105, 110]	> 0.05
KSS knee score pre-operative (median [IQR])	60 [55, 66]	63 [57, 67]	59 [52, 65]	
KSS knee score post-operative (median [IQR])	94 [90, 96]	93 [90, 96]	94 [91, 97]	> 0.05
KSS function score pre-operative (median [IQR])	60 [50, 60]	60 [50, 70]	50 [45, 60]	
KSS function score post-operative (median [IQR])	85 [70, 90]	80 [70, 90]	85 [70, 100]	> 0.05
Re-intervention				
Re-intervention, *N*° (%)	4 (3.13)	2 (3.64)	2 (2.74)	> 0.05
Re-intervention for prosthesis loosening, *N*° (%)	2 (1.56)	1 (1.82)	1 (1.37)	> 0.05
Re-intervention for infections, *N*° (%)	2 (1.56)	1 (1.82)	1 (1.37)	> 0.05
Re-intervention for mechanical failure, *N*° (%)	0 (0.00)	0 (0.00)	0 (0.00)	
Postoperative radiological evaluation				
Radiolucent line, *N*° (%)	6 (4.69)	3 (5.45)	3 (4.11)	> 0.05
Osteolysis, *N*° (%)	1 (0.78)	1 (1.82)	0 (0.00)	> 0.05

*CPE* conventional polyethylene, *HXLPE* highly crosslinked polyethylene, *N*° number of evaluation cases, *p p* value, *PROMs* patient‐reported outcome measures, *ROM* range of motion, *IQR* interquartile range, *KSS* Knee Society Score, *%* percentage

### Re-intervention and post-operative radiological evaluation

There were two re-interventions for infection and two for prosthesis loosening. There were no re-interventions for mechanical failure. In the radiological evaluation, radiolucency lines were evident in six knees, while osteolysis was found only in one CPE TKA (Table 2).

## Discussion

The most important finding of this study is that HXLPE is to be as safe as CPE in TKA, reporting no higher revisions for osteolysis, prosthesis loosening, infections, and mechanical failure. Moreover, higher KSS knee and function scores were found in the HXLPE group than in the CPE, although this difference was not statistically significant.

The increasing number of TKAs implanted globally, and their use in young and active patients has led to the implementation of HXLPE in TKA to improve the prosthesis’s longevity, durability, and performance based on the excellent results obtained in THA [3, 7]. It has been demonstrated that the survival of TKA in younger patients is lower than that in those over 70 years, and this result is probably related to the higher functional demands and subsequent wear of prosthesis components in younger patients [5]. HXLPE in TKA was introduced to overcome these problems; nevertheless, based on the current literature, its use in TKA has reported contradictory results [2, 3, 7]. It should be underlined that the biomechanical properties of the knee are different from those of the hip. The knee is characterised by significant contact stresses and shear forces, with a higher risk of delamination and fatigue fracture than the hip; moreover, the knee has a different pattern of movement of the bearing surfaces, mainly being back and forward, compared to the quasi-elliptical movement of the hip [1, 3, 5]. The excellent results obtained in THA do not imply equal results in TKA. In addition, the higher stresses observed on PE inserts have led to some concerns about using HXLPE in TKA because of its hypothesised inferior mechanical properties compared with CPE and, thus, higher risk of implant failure and need for revision [1–3].

Revision TKA is a complex procedure with a high risk of complications and worse clinical outcomes than primary TKA [2, 13]. The leading cause of early revision is related to infection [14]. Lachiewicz et al. in their study, evaluated the infectious risk with different PE inserts and reported that HXLPE in TKA had a 26% lower risk of revision for infection than CPE [15]. Furthermore, in another 2014 paper, the same authors demonstrated that HXLPE could reduce biofilm formation and bacterial adhesion on the inserts compared with CPE [16]. In long-term follow-up, the leading cause of revision in TKA is aseptic loosening, mainly related to PE wear, debris formation, and implant loosening [17]. Gkiatas et al. in their meta-analysis of national registries, provided evidence that the use of HXLPE resulted in a lower revision rate for aseptic loosening than CPE. However, the overall revision rate between the two types of PE did not differ statistically significantly [8]. Bistolfi et al. in their systematic review and meta-analysis of randomised clinical trials comparing CPE and HXPLE implants, demonstrated no significant differences in radiological and clinical outcomes between the two types of PE [2].

The main concerns in using HXLPE in TKA are related to both the lower mechanical properties of HXLPE compared to CPE and the smaller and hypothetically more biologically active debris particles generated by HXLPE wear [8, 10]. The reduced mechanical properties may be responsible for an increased risk of liners fracture, while debris with higher biological activity may induce a more severe inflammatory response with osteolysis. From a biomechanical viewpoint, HXLPE, compared with CPE, has lower fatigue fracture strength but higher resistance to adhesive and abrasive wear [18, 19]. Previous studies [9, 22] have reported that HXLPE liners could lead to mechanical failure in TKA, because they reduce fracture toughness and increase the risk of liner fracture, as reported by tibial post-fracture cases in posterior stabilised TKA [17, 19]. Nevertheless, these findings were not confirmed by more recent clinical studies in which second-generation, sequentially irradiated, and annealed HXLPE was used, preserving the microstructure without substantially affecting the mechanical properties, which remained similar to those of CPE [2, 11, 20]. Discordant results have been reported regarding the higher risk of osteolysis related to the increased biological activity of wear particles in HXLPE [9, 10]. The size of the debris would seem to play an essential role; particles smaller than 0.05 μm should not activate an inflammatory response, whereas particles larger than 10 μm in diameter may not be phagocytosed. The objective clinical implication of these PE particles with higher biological activity is unclear, and recent meta-analyses provide similar rates of osteolysis between CPE and HXLPE with no significant differences in mid-term follow-up [2, 8].

This paper has several strengths. It is the first study comparing CPE and HXLPE with a minimum follow-up of 10 years. Second, identical prostheses were implanted in both groups except for the CPE and HXLPE liners, and TKAs were performed by the same surgical team with the same surgical technique. This reduced biases due to the characteristics of the prosthetic implants and different surgical techniques that may influence clinical outcomes.

This study also has several limitations. First, given its retrospective nature, it lacks patient randomisation. Although the groups were similar in age, sex, clinical characteristics, and preoperative function, a prospective randomised study would have limited bias and other potentially confounding variables. Second, a posteriorly stabilised design and a mechanical alignment were used in all TKAs, so the results may not apply to different prosthetic designs and other alignment methods. Third, many patients were lost during follow-up leading to a possible selection bias. Fourth, the number of TKAs included and the follow-up period considered may not be sufficient to detect rare or delayed causes of failure, such as polyethylene fracture or osteolysis.

The results reported in this paper observed that HXLPE is as safe as CPE in TKA in a mid-to-long-term follow-up, overcoming concerns regarding its reduced mechanical properties, increased fracture risk and subsequent prosthetic revision. Nevertheless, at the same time, HXLPE demonstrated no significant improvements in clinical and prosthetic revision. Currently, it could not be established that HXLPE improves TKA survival and performance sufficiently to justify the higher costs. Furthermore, the implantation cost is not standardised and fluctuates significantly between geographic areas and manufacturers [5]. Due to these variables, it is difficult to estimate the exact cost difference between HXLPE and CPE. The HXLPE demonstrated less osteolysis, catastrophic fracture absence, reasonable mechanical properties, and long-term survival potential in this retrospective study. Therefore, as reported by other authors [6], the HXLPE could be cost-effective in younger patients to prevent future revisions.

## Conclusion

This study demonstrated that HXLPE is as safe as CPE in mid-to-long-term TKA follow-up; however, there was no significant clinical, radiological, and functional improvement or reduction in revision rates. Therefore, the use of HXLPE in TKA remains controversial.

## Data Availability

The data set analysed in this study is available from the corresponding author on reasonable request.

## Abbreviations

OA: Osteoarthritis
TKA: Total knee arthroplasty
PE: Polyethylene
HXLPE: Highly crosslinked polyethylene
CPE: Conventional polyethylene
IRB: Institutional Review Board
AP: Anteroposterior
ROM: Range of motion
BMI: Body mass index
KSS: Knee Society Score
IQR: Interquartile range
